# Untangling Heterogeneity in Cardiogenic Shock

**DOI:** 10.1016/j.jacadv.2022.100129

**Published:** 2022-10-28

**Authors:** Patrick R. Lawler, Candice K. Silversides

**Figure:**
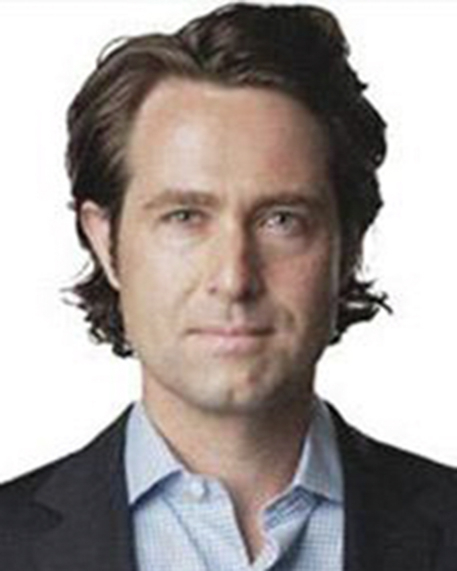

**Figure:**
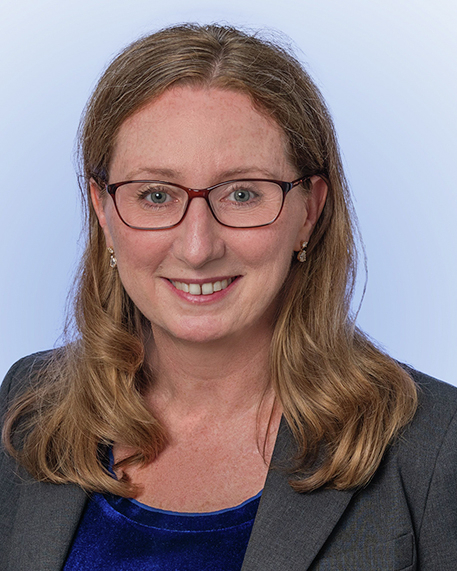

Despite advances in cardiac therapies targeted for cardiogenic shock, mortality remains high. The failure to observe benefit in cardiogenic shock trials is increasingly hypothesized to be due, at least in part, to undercharacterized heterogeneity in the population. Although patient subgroups with distinct pathobiology, prognosis, and response to treatment likely exist within the cardiogenic shock population, it has been unclear how they should be stratified. Emerging methodologies may help identify the clinical and mechanistic traits that stratify this diverse population based on risk, clinical course, and potentially, response to treatment. Important learning from other critically ill patient populations may have relevance to patients with cardiogenic shock.

Machine learning methodologies can integrate large amounts of high-dimensional clinical or molecular data and identify clinical patterns and relevant subgroups of patients.^1^ Unsupervised machine learning examines the data without labels and uses algorithms to identify “hidden” patterns and phenotypes. Using this unconstrained approach, clinical patterns may be identified, some of which may not be apparent even to experienced clinicians. Although machine learning could be useful in many areas of medicine, it may be particularly well suited for studying patients in intensive care units, where large amounts of digitalized data (ie, laboratory data, continuous telemetry recordings, vital signs, and hemodynamic data) are acquired and can be used to identify relevant patterns and subgroups. One such study identified 3 prognostically distinct subgroups of patients with cardiogenic shock based on 6 common laboratory parameters: the noncongested, cardiorenal, and cardiometabolic phenotype, which had the worse prognosis.^2^ Identification of such subphenotypes may allow for improved risk prediction and possibly therapeutic decision making. Additionally, feature selection approaches innate to machine learning methods can be used for biologic discovery and to potentially support drug target development when applied to high-dimensional molecular data in critically ill patients.^3^

In this issue of *JACC: Advances*, a 2-part State-of-the-Art Papers on staging and phenotyping patients with cardiogenic shock describes current approaches to this evidence roadblock.^4^^,^^5^ The reviews provide an overview of how machine learning methods, such as clustering techniques, can be used to improve phenotyping and how subphenotyping using clinical features and biomarkers could help in understanding the pathobiology of diseases and provide a basis for tailored therapy. The identification of subphenotypes could also be used to improve efficiency in clinical trials by enriching study populations with subphenotypes most likely of having an outcome modified by treatment.

Although these are still the early days of machine learning in cardiology, the field is growing rapidly, and applications broadening. Machine learning offers new strategies to advance our understanding of the cardiogenic shock phenotypes and provide more a personalized approach to cardiac care in this population.
